# Sporadic Burkitt Lymphoma Presenting as Refractory Iron-Deficiency Anemia in a Three-Year-Old Boy: A Case Report

**DOI:** 10.7759/cureus.114606

**Published:** 2026-08-16

**Authors:** Natalie R Pettirossi, Nina Simon, George Rogu

**Affiliations:** 1 Pediatrics, New York Institute of Technology, Old Westbury, USA; 2 Pediatrics, RBK Pediatrics, Commack, USA

**Keywords:** intraabdominal mass, iron-refractory iron-deficiency anemia, non-hodgkins lymphoma, pediatric anemia, sporadic burkitt lymphoma

## Abstract

Burkitt lymphoma is a highly aggressive B-cell non-Hodgkin lymphoma that most commonly presents in children with extranodal abdominal disease. Although the diagnosis is often prompted by gastrointestinal symptoms or a palpable abdominal mass, early manifestations may be subtle and nonspecific, delaying recognition. We present the case of a three-year-old boy who was initially evaluated during a routine well-child visit for short stature and found to have microcytic anemia consistent with iron-deficiency anemia (IDA). Eight days after initiating appropriate iron supplementation, he subsequently developed jaundice, a palpable abdominal mass, and progressive clinical decline. Further evaluation with imaging revealed multiple intraabdominal masses, and tissue biopsy confirmed sporadic Burkitt lymphoma with extensive intraabdominal involvement. This case highlights the importance of ongoing clinical reassessment and careful physical examination when patients fail to respond as expected to standard therapy, as seemingly common pediatric conditions may represent the earliest manifestations of an underlying malignancy.

## Introduction

Burkitt lymphoma is an aggressive mature B-cell non-Hodgkin lymphoma characterized by rapid cellular proliferation and dysregulation of the MYC oncogene [1]. Clinically, Burkitt lymphoma is categorized into three variants: endemic, immunodeficiency-associated, and sporadic [2]. Although these variants share similar molecular features, they differ in epidemiology and clinical presentation. In North America and Europe, the sporadic variant predominates and most commonly presents in the pediatric population with extranodal abdominal involvement [3].

Patients with sporadic Burkitt lymphoma often present with rapidly progressive, nonspecific gastrointestinal symptoms such as abdominal pain, distension, nausea, vomiting, or a palpable mass [4]. Given its extremely rapid growth rate, early recognition and prompt initiation of therapy are essential, as Burkitt lymphoma is highly responsive to intensive chemotherapy when diagnosed in a timely manner.

The diagnosis of Burkitt lymphoma is established through a combination of histopathologic, immunophenotypic, and cytogenetic findings. Tumors demonstrate a germinal center B-cell phenotype and are characterized by a nearly 100% Ki-67 proliferation index [4]. Molecular confirmation is typically achieved through identification of a MYC rearrangement by fluorescence in situ hybridization or cytogenetic analysis [5]. Imaging also plays a critical role in evaluation, with ultrasound frequently serving as the initial modality in pediatric patients due to its safety and accessibility. Suspicious findings on ultrasound prompt further assessment with contrast-enhanced computed tomography to better define the extent of disease and to guide tissue biopsy.

Despite these well-established diagnostic criteria, the early manifestations of Burkitt lymphoma may be subtle and nonspecific, posing a diagnostic challenge for providers. This case report describes an atypical presentation of sporadic Burkitt lymphoma that initially manifested as severe iron-deficiency anemia (IDA). While IDA is common in toddlers, failure to demonstrate hematologic improvement within 2-4 weeks of appropriate iron therapy, particularly in the setting of persistent reactive thrombocytosis, should prompt immediate re-evaluation for secondary causes. The case underscores the importance of maintaining a broad differential diagnosis when evaluating persistent or unexplained abnormalities in pediatric patients and highlights the importance of reevaluation and ongoing clinical vigilance when patients do not respond as expected to treatment.

## Case presentation

A previously healthy three-year-old boy presented for a routine well-child examination with parental concern for short stature. The patient was otherwise asymptomatic, with normal activity level and age-appropriate development. Physical examination at that time was notable for height below the expected percentile, without abdominal abnormalities or organomegaly.

Routine screening laboratory evaluation revealed anemia with a hemoglobin of 8.2 g/dL and low mean corpuscular hemoglobin, with normal white blood cell count and thrombocytosis (approximately 528 x 10³/µL). The Mentzer index was calculated at 25, supporting iron deficiency over beta-thalassemia trait. There were no signs of infection or systemic illness, which led to the initial impression of uncomplicated IDA.

The patient was sent for a more complete laboratory evaluation, which demonstrated persistent microcytic, hypochromic anemia with low mean corpuscular hemoglobin, elevated red cell distribution, and continued thrombocytosis. Iron studies were ordered, and the results were consistent with IDA, including serum iron 12 µg/dL, ferritin 6 ng/mL, elevated total iron-binding capacity, and iron saturation 3% (Table 1). Additional evaluation, including thyroid function, celiac screening, hemoglobin electrophoresis, inflammatory markers, and vitamin levels, was unremarkable.

**Table 1:** Laboratory findings from initial presentation through emergency department evaluation MCV: mean corpuscular volume; RDW: red cell distribution width; WBC: white blood cell count; TIBC: total iron-binding capacity; ALT: alanine aminotransferase; AST: aspartate aminotransferase; LDH: lactate dehydrogenase

Test	Initial visit	Follow-up	ED presentation	Reference range
Hemoglobin (g/dL)	8.2	7.2–8.6	8.6	11-13.5
Hematocrit (%)	26.2	-	-	33-39
MCV (fL)	Low	Low	Low	70-86
RDW (%)	Elevated	Elevated	Elevated	11.5-14.5
Platelets (×10³/µL)	528	Elevated	Elevated	150-450
WBC (×10³/µL)	Normal	Normal	-	5-15
Serum iron (µg/dL)	12	-	-	50-120
Ferritin (ng/mL)	6	-	-	7-140
TIBC (µg/dL)	Elevated	-	-	250-400
Iron saturation (%)	3%	-	-	20-50%
ALT (U/L)	-	Normal	484	<40
AST (U/L)	-	Normal	153	<40
Alkaline phosphatase (U/L)	-	Normal	1002	150-420
Total bilirubin (mg/dL)	-	Normal	5.98	<1.0
LDH	-	-	Elevated	-

At this time, the patient remained clinically stable, and the anemia was attributed to iron deficiency. The patient was started on oral ferrous sulfate supplementation with vitamin C, and the family was provided dietary counseling.

Over the following two weeks, the clinical picture began to change. The patient developed new symptoms, including progressive fatigue, decreased appetite, abdominal discomfort, and increasing abdominal distension. On follow-up evaluation, physical examination revealed a firm, distended abdomen with right-sided fullness and a palpable, mobile mass in the right lower quadrant. There was no hepatosplenomegaly appreciated.

Repeat laboratory studies were conducted, which demonstrated persistent anemia (hemoglobin: 8.6 g/dL) with ongoing thrombocytosis and minimal response to iron therapy (Table 1). As the clinical picture started to change, the differential diagnosis broadened. In particular, the abdominal mass warranted further evaluation. An abdominal ultrasound and kidney ureter bladder (KUB) ultrasound were appropriately ordered to further evaluate the mass.

Within 24 hours, the patient clinically deteriorated and presented to the emergency department with worsening abdominal pain, jaundice, anorexia, fatigue, and dark urine. Physical examination revealed scleral icterus and a firm right-sided abdominal mass.

Laboratory evaluation in the emergency department revealed persistent anemia along with markedly elevated liver enzymes (alanine aminotransferase (ALT): 484 U/L; aspartate aminotransferase (AST): 153 U/L), elevated alkaline phosphatase (1002 U/L), conjugated hyperbilirubinemia (total bilirubin: 5.98 mg/dL), and elevated lactate dehydrogenase (724). The patient’s laboratory values now represented a cholestatic jaundice profile (Table 1).

**Figure 1:**
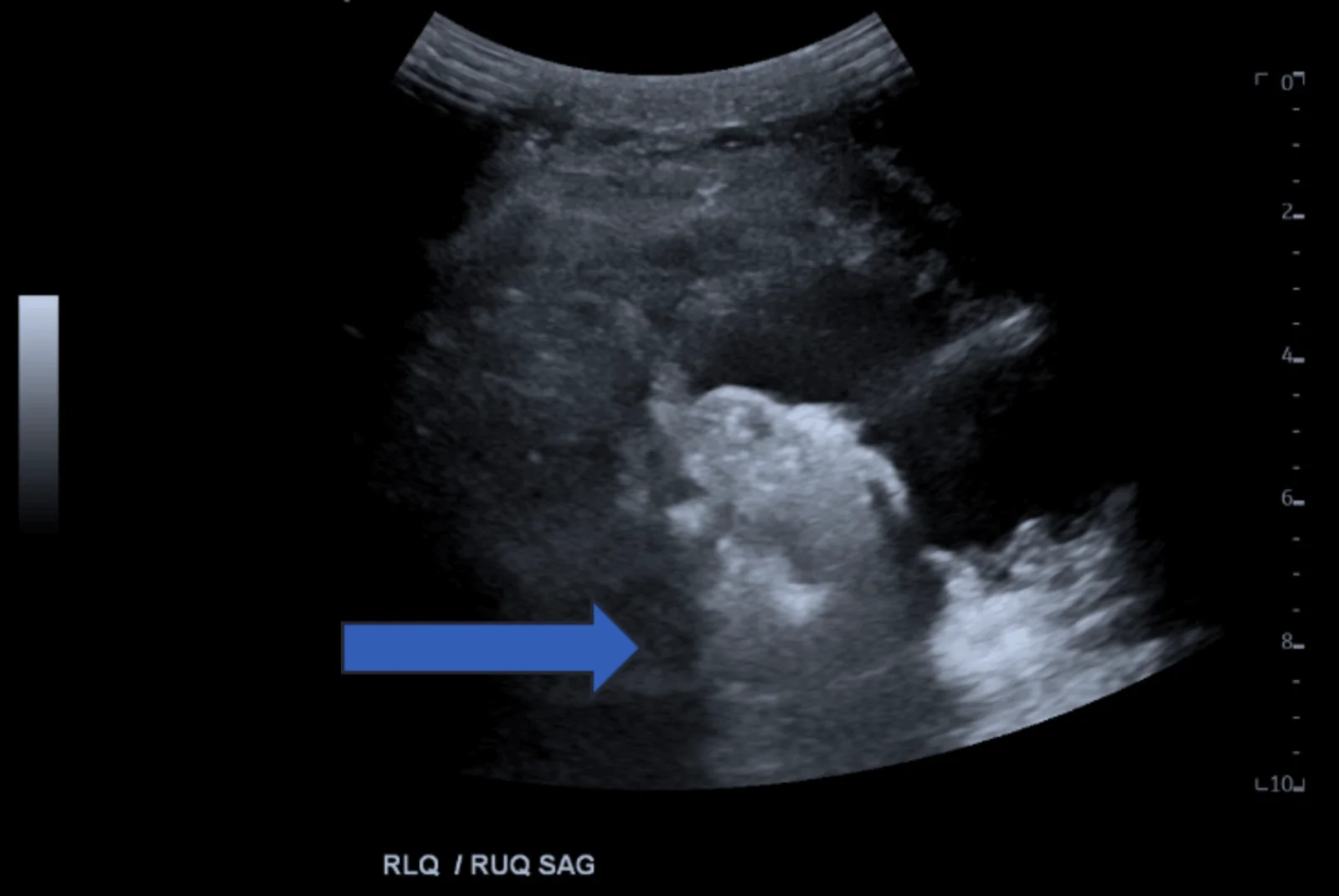
Abdominal ultrasound demonstrating the intraabdominal mass in the right lower quadrant identified during emergency department evaluation RUQ: right upper quadrant; RLQ: right lower quadrant; SAG: sagittal

Abdominal ultrasound in the emergency department revealed a lobulated hypoechoic mass in the porta hepatis, a large mesenteric mass encasing adjacent bowel loops, a mass compressing the renal artery, and another mass in the pelvis region (Figure 1).

**Figure 2:**
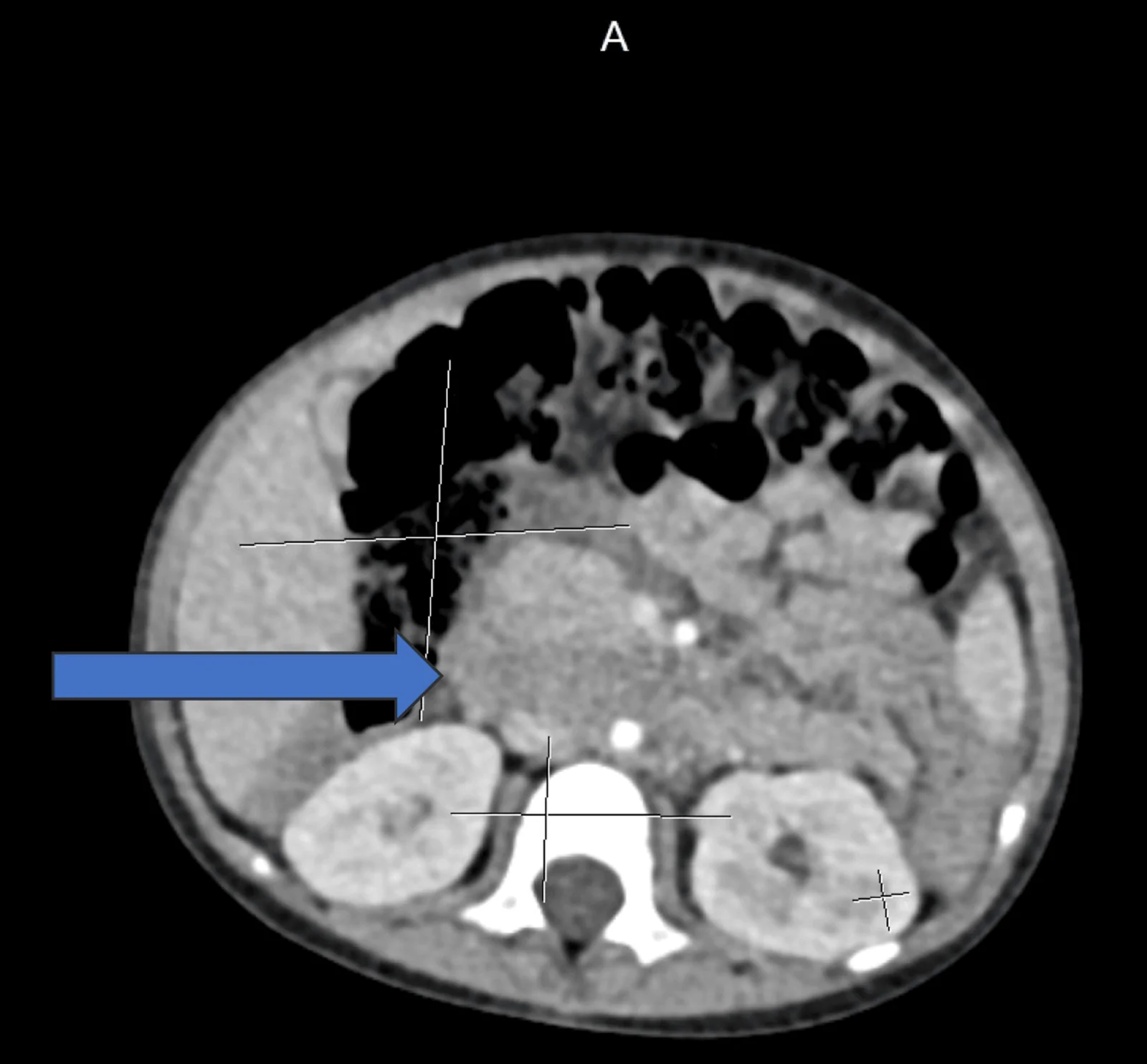
Contrast-enhanced CT scan demonstrating the primary intraabdominal mass during inpatient hospitalization CT: computed tomography

**Figure 3:**
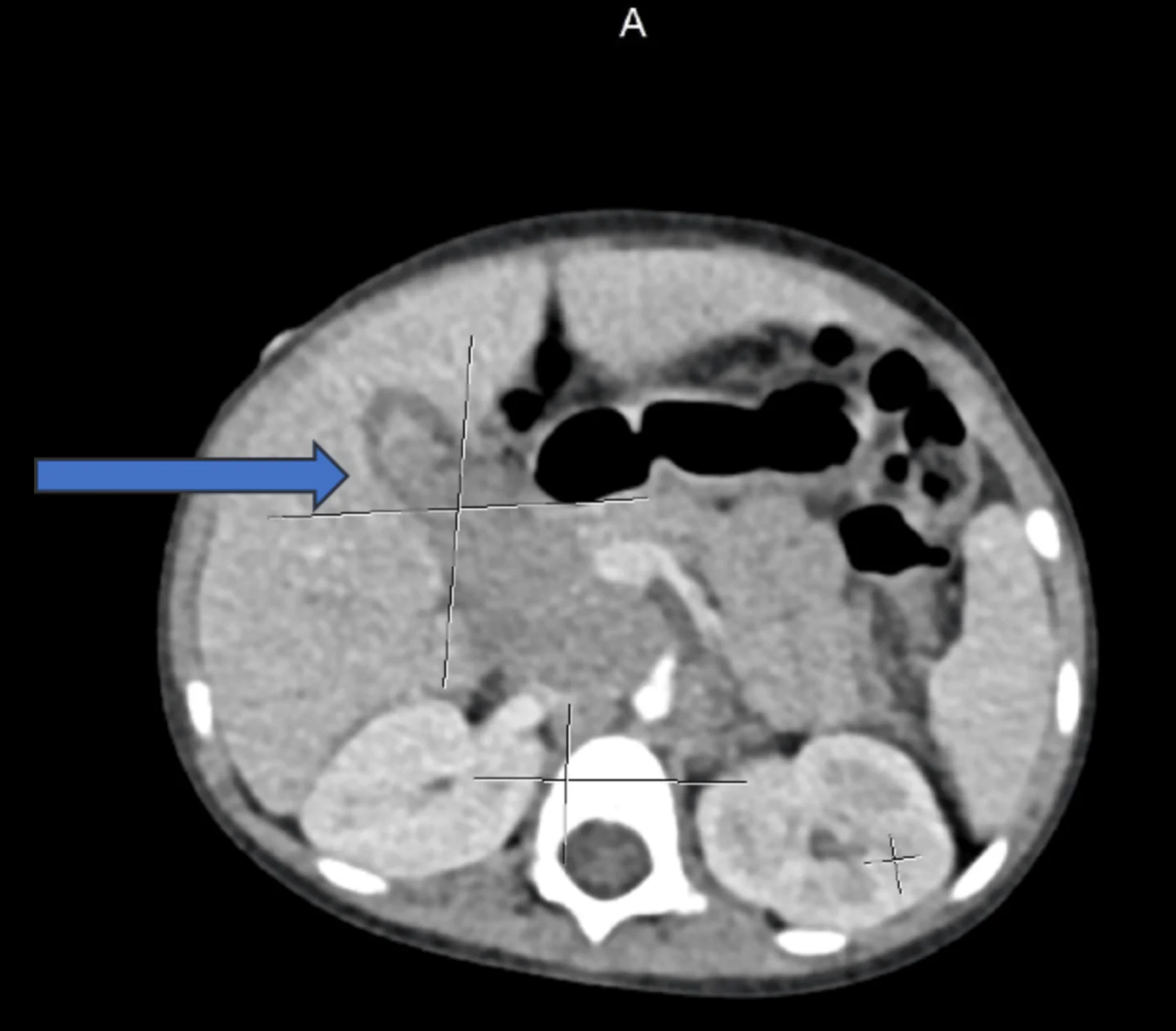
Contrast-enhanced CT scan demonstrating the porta hepatis mass during inpatient hospitalization CT: computed tomography

This prompted the order for a computed tomography (CT) scan of the chest, abdomen, and pelvis (Figures 2-3). The CT findings were highly concerning for malignancy, with radiology suggesting lymphoma, neuroblastoma, or sarcoma. The primary abdominal mass measured 8.8 x 7.0 x 6.6 cm and encased surrounding bowel loops; the porta hepatis mass encased the portal vein, celiac trunk, and right renal artery; the pelvic mass measured 4.8 x 3.0 x 3.0 cm; and the left renal mass was a 1.1 cm enhancing lesion, suggestive of extranodal disease. There was small-volume ascites noted on the CT with clear lungs and no pulmonary metastases.

The patient was transferred emergently to a tertiary care center for further imaging, oncologic evaluation, and tissue diagnosis. The patient was admitted to the hospital and had repeat laboratory work performed (Table 2). 

**Table 2:** Laboratory results during inpatient hospitalization ALT: alanine aminotransferase; AST: aspartate aminotransferase

Laboratory test	Result	Reference range
Lactate dehydrogenase (LDH, peak)	932 U/L	135-225 U/L
Uric acid	3.4 mg/dL	3.4-8.8 mg/dL
Potassium (nadir)	2.6 mmol/L	3.5-5.0 mmol/L
Calcium (nadir)	6.1 mg/dL	8.8-10.8 mg/dL
Phosphorus	3.8 mg/dL	3.6-5.6 mg/dL
Creatinine	<0.20 mg/dL	0.20-0.50 mg/dL
Total bilirubin (peak)	9.6 mg/dL	0.2-1.2 mg/dL
Direct bilirubin (peak)	5.2 mg/dL	0.0-0.3 mg/dL
AST (peak)	251 U/L	10-50 U/L
ALT (peak)	241 U/L	10-50 U/L
Alkaline phosphatase (peak)	1,095 U/L	150-420 U/L
Hemoglobin (nadir)	8.4 g/dL	10.1-15.1 g/dL

Biopsy of the masses confirmed the diagnosis of sporadic Burkitt lymphoma with extensive intraabdominal involvement (Table 3).

**Table 3:** Pathology report of the intraabdominal masses FNA: fine-needle aspiration; IV: intravenous; FISH: fluorescence in situ hybridization; EBV: Epstein-Barr virus; EBER: Epstein-Barr virus-encoded small RNAs

Specimen	Key findings	Interpretation	Clinical significance
Mesenteric mass (FNA + core biopsy)	High-grade B-cell lymphoma	Diagnostic of aggressive lymphoma	Confirms primary malignancy
Immunophenotype	CD20+, PAX5+, CD19+, CD22+, CD79a+, CD38+, HLA-DR+	Mature B-cell phenotype	Consistent with Burkitt lymphoma
Proliferation index	Ki-67 ≈100%	Extremely high proliferation	Hallmark of Burkitt lymphoma
Oncogene expression	C-MYC-positive (diffuse)	MYC-driven malignancy	Supports Burkitt classification
FISH	MYC::IGH fusion-positive	t(8;14)-type rearrangement	Molecular confirmation of Burkitt lymphoma
Negative markers	BCL2-, TdT-, CD5-, CD34-, EBV (EBER)-negative	Excludes other lymphoma subtypes	Helps refine diagnosis
p53 staining	Diffuse positive (suggestive mutation)	Possible TP53 mutation	May indicate higher-risk biology
Bone marrow biopsy	Normocellular marrow, no lymphoma involvement	No marrow disease	Suggests nonstage IV marrow involvement

The patient was admitted to the hospital and initiated on corticosteroid treatment followed by intensive combination chemotherapy and immunotherapy, with close monitoring for tumor lysis syndrome. He demonstrated a rapid and favorable early treatment response, with approximately 50-70% reduction in tumor burden during the initial phase of therapy. This included complete resolution of one of the abdominal masses as well as significant reduction of the others. The patient is expected to continue receiving treatment for the next three months (Table 4).

**Table 4:** Timeline of clinical course RLQ: right lower quadrant; CMP: comprehensive metabolic panel; LFTs: liver function tests; LDH: lactate dehydrogenase; CBC: complete blood count

Date	Encounter	Findings	Evaluation	Outcome
Day 0	Well-child visit	Short stature; asymptomatic	CBC	Anemia identified
Day 2-5	Lab evaluation	Microcytic anemia; low ferritin	Iron studies	Iron-deficiency anemia confirmed
Day 8	Telemedicine	Asymptomatic	-	Iron therapy started
Day 15	Office visit	Fatigue, abdominal distension, RLQ mass	CBC	Poor response to iron therapy
Day 16	ED visit	Pain, jaundice, dark urine, fatigue	CMP, LFTs, LDH	Systemic involvement noted
Day 16	Imaging	Abdominal masses	Ultrasound	Suspicion for malignancy
Day 16	Transfer	-	-	Tertiary care referral
~3 weeks after presentation	Diagnosis	Multifocal disease	Biopsy	Burkitt lymphoma
Weeks 3-6	Treatment	-	Chemotherapy	Therapy initiated
~4 weeks after diagnosis	Follow-up	Clinical improvement	Imaging	Tumor reduction
Months 1-3 after diagnosis	Oncology management	-	-	Continue chemotherapy

## Discussion

This case highlights an atypical presentation of sporadic Burkitt lymphoma that initially manifested as IDA before rapidly progressing to extensive intraabdominal disease within weeks. Although the patient’s initial laboratory findings were consistent with IDA, the subsequent clinical deterioration underscores the importance of ongoing reassessment when the clinical course deviates from the expected response to treatment.

Burkitt lymphoma is a highly aggressive mature B-cell non-Hodgkin lymphoma driven by MYC dysregulation, most commonly resulting from a translocation involving chromosome 8 [1]. This genetic alteration leads to uncontrolled cell growth and rapid tumor expansion. The sporadic subtype typically involves the abdomen, particularly the mesentery and solid abdominal organs [1]. The annual incidence of the sporadic variant is four per one million children younger than 16 years of age, with a median diagnosis age of 30 years [3]. Clinical manifestations are often nonspecific and include abdominal pain, distension, nausea, or a palpable mass. Laboratory abnormalities frequently include elevated lactate dehydrogenase, uric acid, and liver enzymes, reflecting the high tumor burden and rapid cellular turnover of the disease. A defining feature of Burkitt lymphoma is its extremely rapid growth rate, with tumor doubling times estimated at 24 to 48 hours, often resulting in abrupt clinical progression [6].

The most notable aspect of this case is the patient’s presentation with laboratory-confirmed IDA in an otherwise asymptomatic child. Although IDA is common in children and is often attributed to dietary insufficiency, the persistence of severe anemia despite iron supplementation, accompanied by continued thrombocytosis, prompted further evaluation. Thrombocytosis may occur as a reactive response to severe iron depletion but may also reflect systemic inflammation associated with malignancy. In this case, the thrombocytosis was likely multifactorial, with contributions from iron deficiency and malignancy-associated inflammation. Inflammatory cytokines such as interleukin-6 can increase hepcidin production, promoting iron sequestration and contributing to anemia of inflammation; however, without inflammatory markers or hepcidin measurements, the relative contribution of these mechanisms cannot be determined. The lack of expected hematologic improvement despite treatment ultimately served as an important clinical clue to an underlying process beyond uncomplicated iron deficiency.

As the patient developed new systemic and gastrointestinal symptoms, such as fatigue, anorexia, distension, and the finding of a mass, the differential diagnosis was appropriately broadened. These findings are not characteristic of uncomplicated IDA and cannot be explained by side effects of iron supplementation either. The rapid evolution of symptoms over a short time period is consistent with the known biology of Burkitt lymphoma. This case demonstrates the importance of recognizing when new symptoms or physical exam findings are disproportionate to an established diagnosis and warrant further evaluation.

Imaging played a critical role in establishing the diagnosis. Following the change in clinical status, abdominal ultrasound identified both a porta hepatis mass and a large mesenteric mass encasing bowel loop, findings highly concerning for malignancy. Ultrasound is the preferred initial imaging modality in pediatric patients due to the absence of ionizing radiation. However, cross-sectional imaging with computed tomography is typically required for comprehensive staging and assessment of disease extent [7]. Burkitt lymphoma commonly presents as bulky abdominal masses that may surround adjacent structures without causing early bowel obstruction, potentially delaying recognition until significant disease burden has occurred [3].

Definitive diagnosis requires tissue biopsy with histopathologic, immunophenotypic, and cytogenetic evaluation. Current World Health Organization classification emphasizes the integration of genetic features, morphology, and the immunophenotype to distinguish Burkitt lymphoma from other high-grade B-cell lymphomas [5]. Tumors characteristically demonstrate a germinal center B-cell phenotype, including expression of CD10, CD20, and BCL6, absence of BCL2, and a Ki-67 proliferation index approaching 100% [8]. Molecular confirmation of MYC rearrangement with fluorescence in situ hybridization or cytogenetic analysis further supports the diagnosis. Prompt tissue diagnosis is critical because early initiation of therapy significantly impacts clinical outcomes.

Treatment consists of intensive, multiagent chemotherapy regimens with central nervous system prophylaxis and frequently incorporates rituximab in CD20-positive disease [9]. These patients are at an increased risk of tumor lysis syndrome due to the high proliferation rate of the tumor, and as such, prophylaxis with aggressive hydration, rasburicase, or allopurinol is often provided [7]. Despite its aggressive nature, Burkitt lymphoma is highly chemosensitive, with cure rates in pediatric populations approaching 90% with appropriate treatment [10]. The rapid and significant tumor reduction observed in this patient is consistent with the expected treatment response.

Previous reports have described Burkitt lymphoma presenting with atypical features that mimic more common pediatric conditions, including appendicitis, gynecologic disease, and other benign gastrointestinal disorders [11]. These reports, together with our case, reinforce the importance of maintaining a broad differential diagnosis when symptoms evolve unexpectedly. Clinicians must remain vigilant for "red flags," such as lack of response to treatment, progression of symptoms, or development of new findings that are inconsistent with the presumed diagnosis.

This case report is limited by its single-patient design, which restricts the generalizability of the findings to the broader population of patients with sporadic Burkitt lymphoma. It still provides valuable insight, however, into an uncommon presentation that may aid in earlier recognition of similar cases.

In summary, this case emphasizes the need for ongoing clinical reassessment in pediatric patients with presumed IDA who fail to respond as expected or develop new symptoms. Careful attention to changes in the clinical picture, particularly emergence of systemic or abdominal findings, may facilitate earlier recognition of aggressive malignancies such as Burkitt lymphoma. Given the tumor’s rapid growth rate and excellent response to timely treatment, early diagnosis remains critical for achieving favorable outcomes.

## Conclusions

This case demonstrates how sporadic Burkitt lymphoma may initially present with seemingly benign symptoms, including isolated IDA, before rapidly progressing to overt malignancy. The diagnosis was ultimately established after the patient’s clinical course evolved in a manner inconsistent with uncomplicated IDA, prompting further evaluation and revealing an underlying malignancy. Clinicians should maintain a broad differential diagnosis when common pediatric complaints fail to follow an expected clinical course, as early identification and treatment of Burkitt lymphoma can significantly impact patient outcomes.
